# P-1149. Reducing Central Line-Associated Bloodstream Infections (CLABSI) Using Bundled Care Strategies: A 3-Year Follow-up Study

**DOI:** 10.1093/ofid/ofaf695.1343

**Published:** 2026-01-11

**Authors:** Leena James, Jomel Raju

**Affiliations:** St. Joseph's College of Pharmacy, Elampally, Kerala, India; St. Joseph's College of Pharmacy, Cherthala, Pala, Kerala, India

## Abstract

**Background:**

Central Line-Associated Bloodstream Infections (CLABSIs) significantly increase morbidity, mortality, and healthcare costs, especially in resource-limited settings. Bundled care strategies have demonstrated efficacy in high-income settings, but evidence from secondary care hospitals in India remains scarce. This study evaluates the impact of implementing a CLABSI prevention bundle over a 3-year period in a tertiary-level hospital in rural South India.

**Methods:**

A prospective, quasi-experimental study was conducted from January 2022 to December 2024 in a 12-bed adult ICU. The intervention included strict adherence to a CLABSI prevention bundle: hand hygiene, maximal sterile barrier precautions, chlorhexidine skin antisepsis, optimal catheter site selection, and daily assessment of line necessity. Baseline CLABSI rates were recorded for 6 months prior to intervention. Data were analyzed using Poisson regression and χ² tests for significance.

**Results:**

At baseline, the CLABSI rate was 8.9 per 1000 catheter-days. Following bundle implementation, the rate declined to 5.1 in year 1, 2.8 in year 2, and 1.7 in year 3 (p < 0.001). Overall, there was a reduction of 80.9% in CLABSI rates over 3 years. Hand hygiene compliance improved from 58% to 91% (p < 0.001), and adherence to full bundle components increased from 42% to 87% (p < 0.001). The intervention prevented an estimated 49 bloodstream infections, translating to approximately 490 ICU bed-days saved and a cost saving of INR 28.6 lakh (∼USD 34,400).

**Conclusion:**

Implementing bundled care strategies in a resource-limited Indian ICU substantially reduced CLABSI rates and healthcare costs. Sustained leadership support, nurse-driven audits, and ongoing training were critical to success.

**Disclosures:**

All Authors: No reported disclosures

